# The Different Faces of the TDP-43 Low-Complexity Domain: The Formation of Liquid Droplets and Amyloid Fibrils

**DOI:** 10.3390/ijms22158213

**Published:** 2021-07-30

**Authors:** Hung-Ming Chien, Chi-Chang Lee, Joseph Jen-Tse Huang

**Affiliations:** 1 Institute of Chemistry, Academia Sinica, Nangang, Taipei City 115, Taiwan; sheffieldchien@gate.sinica.edu.tw (H.-M.C.); celegans@gmail.com (C.-C.L.); 2 Department of Chemistry, National Taiwan University, Taipei City 115, Taiwan; 3 Chemical Biology and Molecular Biophysics Program, Taiwan International Graduate Program, Academia Sinica and National Taiwan University, Taipei City 115, Taiwan; 4 Department of Applied Chemistry, National Chiayi University, Chiayi City 600, Taiwan; 5 Neuroscience Program of Academia Sinica, Academia Sinica, Taipei City 115, Taiwan

**Keywords:** amyotrophic lateral sclerosis, TAR-DNA binding protein 43, low-complexity domain, liquid droplet, amyloid fibrils

## Abstract

Transactive response DNA-binding protein 43 (TDP-43) is a nucleic acid-binding protein that is involved in transcription and translation regulation, non-coding RNA processing, and stress granule assembly. Aside from its multiple functions, it is also known as the signature protein in the hallmark inclusions of amyotrophic lateral sclerosis (ALS) and frontotemporal lobar degeneration (FTLD) patients. TDP-43 is built of four domains, but its low-complexity domain (LCD) has become an intense research focus that brings to light its possible role in TDP-43 functions and involvement in the pathogenesis of these neurodegenerative diseases. Recent endeavors have further uncovered the distinct biophysical properties of TDP-43 under various circumstances. In this review, we summarize the multiple structural and biochemical properties of LCD in either promoting the liquid droplets or inducing fibrillar aggregates. We also revisit the roles of the LCD in paraspeckles, stress granules, and cytoplasmic inclusions to date.

## 1. Introduction

Transactive response DNA-binding protein 43 (TDP-43) is a nucleic acid-binding protein that is involved in RNA processing and is essential for the development of the central nervous system [1,2]. While many studies have elucidated the pivotal roles of TDP-43 in multiple cellular functions, emerging studies have also uncovered its pathological roles after it was identified as the long-sought culprit of the ubiquitinated and hyperphosphorylated hallmark inclusions in amyotrophic lateral sclerosis (ALS) and frontotemporal lobar degeneration (FTLD) patients [3,4,5,6]. Recently, it has been demonstrated that TDP-43 could form liquid droplets via its low-complexity domain (LCD) and thereby mediate the stress granule assembly [7]. Meanwhile, accumulating evidence has also suggested the LCD has a strong propensity to form amyloid fibrils and pathological inclusion bodies [8], hinting at its versatile faces.

**Figure 1:**
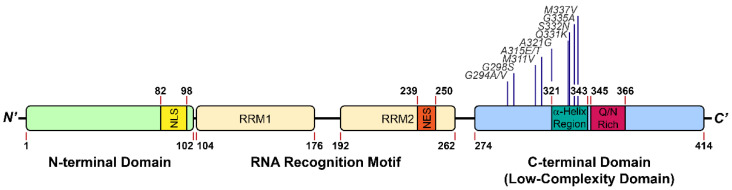
Structure feature of TDP-43. The pathological mutations highlighted in this review are included.

TDP-43 contains 414 amino acids and comprises four domains, including an *N*-terminal domain (NTD, amino acid (aa) 1–102) associated with TDP-43 dimerization [9], two RNA recognition motifs (RRM1: aa 104–176; RRM2: aa 192–262) that bind to nucleic acids [10,11], and a C-terminal low-complexity domain (LCD) (aa 274–414) (Figure 1) [12]. With the nuclear localization signal (NLS) and the nuclear export signal (NES), TDP-43 can shuttle between the nucleus and cytoplasm. Under physiological circumstances, TDP-43 is primarily localized in the nucleus [13], then is relocated to the cytoplasm and self-aggregates into insoluble inclusions in diseases [14]. TDP-43 forms homodimers through the NTD, which have enhanced pre-mRNA splicing activity and prevent cytoplasmic TDP-43 from self-aggregation [15]. Apart from NTD, it has been shown that the LCD could also stabilize the homodimers of TDP-43 through weak self-interactions [16].

As an RNA binding protein, TDP-43 contains two highly conserved RNA recognition motifs (Figure 1), which preferentially bind to the UG/TG-rich sequences of RNA/DNA molecules [10,11]. Through X-ray crystallography, it was demonstrated that both RRMs in TDP-43 dimers participated in the nucleic acid-binding and worked cooperatively to achieve their high affinity and specificity [11]. By applying individual nucleotide-resolution UV cross-linking and immunoprecipitation (iCLIP), Tollervey et al. demonstrated that TDP-43 interacted with introns and 3′-untranslated regions of mRNAs, and non-coding RNAs, hinting at its possible roles in regulating RNA splicing [5]. Additionally, TDP-43 is also involved in neuronal survival or development through regulating the integrity and maturity of mRNAs [17]. Until now, the reported functions of TDP-43 include transcription and translation regulation, messenger RNA (mRNA) splicing, mRNA transportation, mRNA stabilization, mRNA maturation, long non-coding RNA processing, and stress granule assembly [5,12]. Moreover, TDP-43 binds to its own mRNA 3′-untranslated region sequence to regulate its own expression level through a negative feedback loop [18], which is necessary for cell survival for either deficiency or overexpression of TDP-43 induces cytotoxicity both in vitro and in vivo [17,19,20]. Deletion of the LCD abolishes the autoregulation and RNA splicing activity of TDP-43, which highlights the essential role the LCD plays in TDP-43 function.

## 2. The Introduction of TDP-43 LCD

### 2.1. The Structure of TDP-43 LCD

The LCD is the stretch of protein with unique amino acid composition and disordered conformation [21]. In spite of its ubiquitous presence in the realm of protein, attention has been sufficiently paid to its structural and functional characterization in the past. However, emerging evidence have shown that some LCDs are capable of forming secondary structures and play an important role in a variety of cellular functions [9,16,22]. Computational and experimental studies indicate LCD can mediate the protein–protein interactions, which in turn regulate gene expression [23,24,25,26]. From the insights gained by structural analyses, LCD mediates protein solubility and engages in the liquid–liquid phase separation (LLPS) [27,28,29]. In addition, LCD forms amyloid fibrils, thereby contributing to disease pathogenesis [30,31,32,33,34]. Since LCD is polymorphic under different environments, understanding how LCD transits from its native state to liquid droplets or amyloid fibrils should inform us on the underlying molecular mechanism of proteinopathy in these diseases.

**Figure 2:**
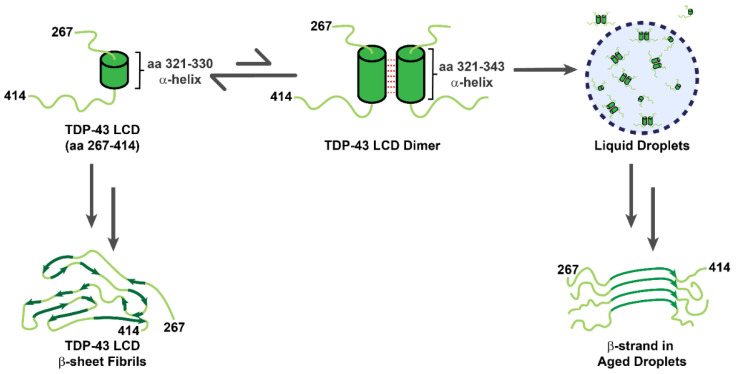
The polymorphic nature of TDP-43 LCD. TDP-43 LCD contains a α-helical structure which promotes the dimerization of TDP-43 LCD and formation of liquid droplets. Upon incubation, TDP-43 LCD liquid droplets transforms into fibrils.

The LCD of TDP-43 is located at its C-terminus (mostly spanning the stretch of aa 267–414) (Figure 1) and includes glutamine/asparagine (Q/N)- and glycine (G)-rich regions. According to the Predictor of Natural Disordered Regions software (PONDR), 80% of TDP-43 LCD is structurally disordered [35]. However, a short region of aa 321–330 recently has been identified to assume an α-helical structure using nuclear magnetic resonance (NMR) spectroscopy [16]; this structure can induce dimerization through an intermolecular helix–helix contact. Moreover, the helical content of each LCD was enhanced during the dimerization according to the molecular simulation and NMR analysis (Figure 2) [36]. Conicella et al. further suggested the intermolecular helix–helix contact promoted the TDP-43 liquid droplet, in which the pathological mutation in LCD (A321G) significantly reduced the helical signature of the LCD and decreased its liquid–liquid phase separation (LLPS) propensity [16,36]. By contrast, another ALS-related mutation (G335D) in the LCD raised the helix content and thereby increased LLPS.

While an α-helix structure has been identified in the LCD, recent literature reported other secondary structure also existed in this domain under different situations (Figure 2) [36,37]. Within these cases, Fonda et al. showed aged TDP-43 liquid droplets gradually transformed into β-strand-rich fibril through stabilization by the segment of TDP-43_365–400_ [36,37]. Under a mildly acidic condition (pH 4), Li et al. showed through cryogenic electron microscopy (cryo-EM) that the LCD could form amyloid fibrils with a core architecture containing 14 β-strands linked by rigid turns and loops [34]. The aforementioned results demonstrate the polymorphic structures that the LCD of TDP-43 may assume in solution or LLPS, while they mostly adopt β-strand conformation in fibrils.

### 2.2. The Possible Function of TDP-43 LCD

Despite being a site for more than 50 ALS-causing mutations and its importance in the etiology of diseases [38], the physiological role of TDP-43 LCD remains largely obscure. In this regard, delineating its interacting proteins has become an urgent issue. As a member of the heterogeneous nuclear ribonucleoproteins (hnRNPs), TDP-43 binds to hnRNP A1/A2 and hnRNP B1 in the presence of LCD to induce cooperative nucleic acid splicing [39,40]. Additionally, D’Ambrogio et al. further showed that TDP-43 interacted with hnRNP A2 through the segment of TDP-43_321–366_ for this splicing regulation [36,41]. Furthermore, TDP-43 LCD (218–414) interacts with both wild-type and the mutant ubiquitin-like protein ubiquilin-2 (UBQLN2) in the inclusion bodies of cells [42,43], and Cassel et al. confirmed this binding and showed its crucial role in the clearance of both TDP-43 and TDP-43 LCD (aa 170–414) [44]. These results demonstrate the importance of UBQLN2 in the pathogenesis of ALS. Although the current understanding of the LCD function is still limited, a growing body of literature has indicated TDP-43 LCD can form either liquid droplets or fibrils in response to different environments, highlighting its two faces in the physiology and pathology.

## 3. The Introduction of TDP-43 LCD

### 3.1. TDP-43 LCDs Form Liquid Droplets

Recently, demixing liquid droplets (also known as biomolecular condensates) through liquid–liquid phase separation (LLPS) between biomacromolecules has been considered as one of the underlying mechanisms for the formation of many critical membraneless organelles in cells, including nucleoli, Cajal bodies, paraspeckles, and stress granules [45,46,47]. Given their liquid-like property, droplets exhibit flexible and dynamic internal structures and can exist for hours or even days despite their extremely fast process of assembly and disassembly [45,48,49]. Though the interior of droplets is separated from the outside environment, the content material in different droplets could still be exchanged through passive diffusion, as well as fusion and fission processes [47,50]. It has been suggested that the driving force behind LLPS is to reduce the free energy of the whole system by forming energetically favorable interactions between biomacromolecules, such as π–π stacking, dipole–dipole interactions, cation–π interactions, and charge–charge interactions [51]. Since most of these interactions are relatively weak, these droplets are sensitive to changes in temperature and ionic strength [45,47,50]. Consequently, post-translational modifications of proteins can act as a switch to regulate LLPS and influence their physiological functions by altering their net charges [52]. For instance, protein phosphorylation can initiate and fine-tune the assembly or disassembly of liquid droplets in vivo [53,54,55,56]. Moreover, acetylation and methylation can also affect the cation–π or charge–charge interactions to modulate LLPS [57,58,59]. Although the components within these liquid droplets in vivo are complicated, proteins with LCDs, TDP-43 included, were extensively found within, implying LCDs play an essential role in mediating droplet formation [27,28,48,60,61,62].

As a major component of several membraneless granules such as paraspeckles and stress granules, TDP-43 is suspected to regulate the assembly and function of these organelles [63,64,65,66,67]. Although these intracellular spherical granules appear indistinctive from the disease-related pathological inclusions under conventional microscopes, they have very different properties from the latter. In this regard, fluorescence recovery after photobleaching (FRAP) was used to assess the fluidity of the granules. By leveraging LLPS principles, TDP-43-positive granules are able to assemble and disassemble quickly, and aptly exchange material with the outside environment. In rodent primary cortical neurons, the TDP-43-positive ribonucleoprotein granules exhibited liquid-like properties and mediated several cellular functions [68]; however, ALS-linked mutations, such as G298S and M337V, significantly decreased their motility and increased their viscosity, which led to the impairment of their normal function to transport granules (Figure 1) [68].

In spite of its abundance in stress granules and paraspeckles, how TDP-43 participates in the assembly of cellular granules remains obscure. Recent studies revealed TDP-43 promoted liquid-droplet formation through the intermolecular interactions between its LCDs [7,69,70]. TDP-43 LCD is capable of mediating LLPS at low temperature and high salt concentration in the absence of a molecular crowding agent (e.g., polyethylene glycol) [16,37,71]. As mentioned above, the α-helical structure of the segment of TDP-43_321–343_ in the TDP-43 hydrophobic core is essential for the droplet formation (Figure 2), and many mutations in this region decrease the fluidity of LLPS or even fail to induce LLPS [36,71,72]. While the α-helix holds the key to the initiation of dimerization and LLPS in these cases, the aromatic amino acid residue (e.g., tryptophan) in TDP-43 LCD serves as the main driving force to stabilize LLPS, as it provides additional hydrophobic interactions [73]. Disrupting hydrophobic interactions by urea or 1,6-hexanediol inhibits the formation of droplets, evincing the importance of hydrophobic interactions for TDP-43-dependent droplet formation [71,74]. Li et al. showed that LCD with W334G/W385G/W412G mutations failed to induce LLPS under the same condition despite their similar α-helix content deduced from secondary chemical shift analysis in NMR [73]. On the other hand, the ALS-related mutants (M337V and Q331K) altered the LLPS propensity with a neglectable impact on its helical structure [74]. Besides TDP-43_321–343_, another core region, TDP-43_365–400_, was found by NMR to form β-strands and stabilize the liquid-droplet structure by closed alignment (Figure 2) [37]. Interestingly, TDP-43 LCD liquid droplets have been shown to be thioflavin-S-positive [75], thus the interior of these droplets is likely rich in closely packed β-strands, which allows binding of this fluorescent dye (also see below). It was reasonable to hypothesize that the intermolecular contact between the segment of TDP-43_311–360_ and/or the segment of TDP-43_365–400_ underlies the formation of liquid droplets and amyloid fibrils [16,36,37,76]. Although the detailed secondary structures of these two segments vary among different studies [16,36,37,76], these structural differences could result from differences in protein preparation, buffer condition, and measurement.

In addition to conformation and interaction, the net charge of TDP-43 LCD and the salt concentration in the buffer also influences the process of LLPS [71,74]. Li et al. suggested the net charge of the TDP-43 LCD inhibited LLPS, while its hydrophobicity promoted the assembly of the liquid droplet. Babinchak et al. showed that LCD was prone to undergo LLPS when the repulsion force by the predicted net charge decreased in a neutral pH environment [71]. When salts were added into the LCD solutions, they neutralized the charge and counteracted the electrostatic repulsion between the LCDs, and thus promoted LLPS [71,74]. While all of these results evinced that TDP-43 LCD could undergo LLPS and form liquid droplets without the assistance of other proteins or nucleic acids in the experimental environments in vitro, the situation is likely to become much more complicated when similar studies are attempted in cells, for high salt concentration and a low temperature microenvironment are rarely found in cells. It is also noteworthy to mentioned that these in vitro experiments usually demonstrated at a concentration around the micromolar range. However, the deduced amount of TDP-43 in a cell is around 10^−6^~10^−4^ pmole [77], and the resulting concentration is lower than the concentration practiced in in vitro liquid-droplet experiments. Moreover, in addition to LCD, other domains in TDP-43 also contributed to the LLPS process.

**Figure 3:**
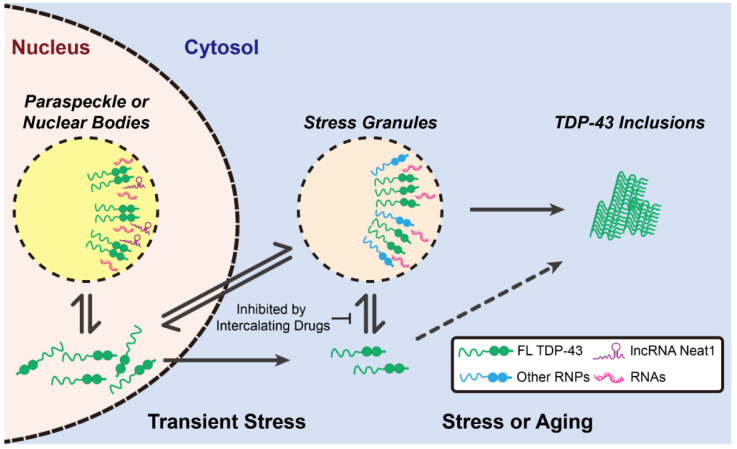
The distinct roles of TDP-43 condensates. Upon transient stress, the disperse TDP-43 binds to long non-coding RNA Neat1 to form sheltering nuclear bodies. Meanwhile, cytosolic TDP-43 can also form stress granules, along with other RNPs and RNAs. Upon prolong stress or aging, stress granules might proceed toward the maturation process and transform into inclusions.

In 2018, Wang et al. found TDP-43 *N*-terminal domain (NTD) also influenced LLPS in cells through the head-to-tail self-interaction of TDP-43 NTD-promoted TDP-43 dimerization [78]. Additionally, the phosphomimetic mutant in NTD (S48E) disrupted TDP-43 self-assembly and prevented droplet formation, revealing that the phosphorylation of NTD is involved in the process of the droplet formation [78]. Collectively, it was surmised that the head-to-tail polymerization of NTD, together with the helix–helix contact between LCD, initiates and stabilizes the TDP-43 LLPS [72,78,79]. Meanwhile, several studies also pointed out the RNA-binding motifs in TDP-43 bind to RNA and promote liquid-like granule formation in cells (Figure 3) [65,80,81,82]; when the interaction between the RNA-binding motifs and RNA is abolished, the assembly and function of TDP-43-associated membraneless organelles is accordingly disrupted. In 2019, Fang et al. displayed a class of small-molecule-based nucleic acid intercalators that inhibited TDP-43-mediated formation of stress granules and subsequently prevented the formation of ALS-associated protein aggregates (Figure 3) [83]. In the presence of these inhibitors, the accumulation of cytoplasmic TDP-43 inclusions was reduced in the differentiated motor neurons from the ALS patient-derived induced pluripotent stem cells (iPSCs). In contrast, by optogenetic strategy, Mann et al. showed the pathological inclusions formed outside the stress granules (Figure 3) [84]. They hypothesized that RNA binding further inhibited the LCD self-assembly and thus prevented the formation of pathological inclusions (Figure 3) [84]. In addition to cytosolic assembly, nuclear organelles (e.g., paraspeckles) also shelter TDP-43 by forming liquid droplets with a long non-coding RNA, nuclear-enriched abundant transcript 1 (NEAT1) (Figure 3) [85]. Interestingly, apart from the RNA-binding motifs, the LCD also has potential to interact with RNA [16]. Although the LCD alone is sufficient to form liquid droplets, addition of RNA further enhances LLPS through the LCD–RNA interactions [16]. Since TDP-43 LCD is rich in hydrophobic and charged amino acid residues, the hydrophobic and electrostatic interactions between RNA and the LCD may be the main driving force for LLPS. Other than RNA, TDP-43 may also collaborate with other proteins (e.g., HSP70) to undergo LLPS [86]. More recently, Yu et al. indicated that the acetylation of TDP-43 drove its LLPS to form a uniquely ring-shaped structure within the cell nucleus [86]. Furthermore, they also found this intranuclear liquid spherical annuli (iLSA) is an anisotropic structure, suggesting its ordered liquid property. Interestingly, iLSA (also termed as “anisosome”) is independent of RNA, as both NTD and LCD were required for this liquid spherical shell structure. Through proteomic analysis, they found that HSP70 chaperone family proteins comprise the core of iLSA. As iLSA structure is susceptible in response to HSP70 inhibitor, it seems HSP70 actively maintain the specialized layer of TDP-43 structure. This work provided strong evidence that formation of TDP-43 LCD-mediated LLPS is also under the governance of chaperones.

### 3.2. TDP-43 LCDs Form Amyloid Fibrils

In addition to its newly emerging role in liquid droplets, the LCD has long been known to confer LCD-containing proteins with the propensity to form amyloid fibrils [27,30,87]. Despite the fact that amino acid composition of these proteins varies, amyloid fibrils formed by misfolded proteins or peptides share a few salient features, such as β-sheet secondary structure, interaction with amyloid-sensitive dyes (Congo Red, Thioflavin S, and Thioflavin T), and the tendency to self-aggregate [88,89]. Currently, the protein-folding funnel hypothesis has been proposed to explain how functional proteins eventually are trapped into misfolded states. Within these cases, the intramolecular interactions guide the folding process of a nascent chain into its functional native state that enables the energy of the entire system to stay at a local minimum [90]. Once a protein is partially misfolded, the intermolecular interactions may override the intramolecular interactions, which in turn lead to accumulation of misfolded proteins and high-order assembly of proteins with a β-sheet signature. These high-ordered aggregates, ranging from oligomers and protofibrils to amyloid fibrils, may have different conformations with distinctly local minima on the energy landscape. In this model, amyloid fibrils assume a conformational state with the lowest minimum energy on the landscape. In order to decrease the free energy, the polypeptide chain folds toward the amyloid fibril state during the refolding process [91,92,93,94]. Although many studies have characterized the biochemical and biophysical properties of amyloid oligomers and fibrils [95,96,97], their detailed mechanism and role in proteinopathies remain inconclusive [98].

**Figure 4:**
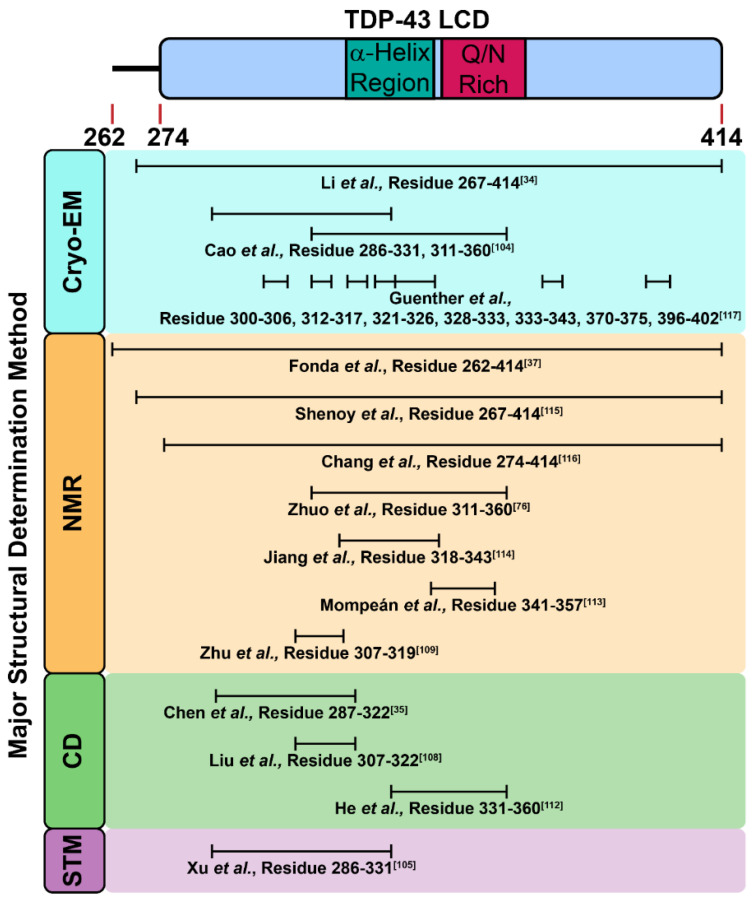
Reported TDP-43 LCD segments and fragments with amyloidogenic properties.

As a pathological hallmark in ALS, cytoplasmic TDP-43 inclusions were mainly described as amorphous aggregates that were thioflavin-negative; however, a subset of diagnostic skein-like inclusions were thioflavin-positive, consistent with the ability of TDP-43 to deposit into amyloid fibrils [99,100,101]. Given the fact that the amino acid sequence and composition of the LCD of TDP-43 shares similarities with prion proteins (namely prion-like properties), a working hypothesis argues that the LCD is responsible for TDP-43 aggregation [34,102,103,104]. The insights from solution and solid-state NMR spectroscopy suggest the segment of TDP-43_311–360_ might engage in a structural transformation from α-helix to parallel β-sheet during incubation (Figure 4) [76]. Along this direction, the cryoEM-reconstructed structure of TDP-43_311–360_ revealed its intrinsically polymorphic nature of amyloid fibrils [34]. In addition to this segment, TDP-43_286–331_ also assembled into Thioflavin-T-positive fibrils (Figure 4) [105,106]. Moreover, pathological mutants (A315pT and A315E) of TDP-43_286–331_ further enhanced aggregation propensity due to the electrostatic interaction between Arg293 and negative charge from phosphorylated-Tyr315 or Glu315.

Due to the poor solubility of the LCD, the purification of its recombinant protein is challenging. Thus, applying chemically synthesized peptides to mimic the fragments of the LCD is a feasible strategy to examine its amyloidogenicity and their biophysical properties in different environments. In fact, as of 2010, we had synthesized different peptide fragments in the LCD, and reported that its D1 segment (TDP-43_287–322_) was able to form cytotoxic amyloid fibrils with strong a β-sheet signature in vitro (Figure 4) [35]. The two D1 pathological mutants (G294A and A315T) exhibited faster fibril formation kinetics and enhanced Thioflavin-T-binding compared with D1. Later, Sun et al. showed the detailed biophysical properties of D1 segments with more pathological mutations, and displayed that the glycine-to-proline replacement (TDP-43_287–322_GGG308PPP) abolished the amyloidogenesis of the D1 segment [107]. Through molecular-dynamics simulations, Chen et al. further showed D1 formed oligomers and induced membrane pore formation in vitro, which implied the possible role of D1 in membrane disruption [108]. In 2013, through further truncation, Liu et al. delineated an even shorter core sequence in the D1 segment (TDP-43_307–322_) with amyloid properties, and showed that TDP-43_307–322_ transformed from random coil to β-sheet after incubation (Figure 4) [109]. Moreover, TDP-43_307–322_ was capable of disrupting artificial lipid membrane and acting as “seeds” to accelerate the aggregation process of full-length TDP-43 [108,109]. After a year, Zhu et al. reported TDP-43_307–319_ fragments adopted an anti-parallel β-sheet conformation by intermolecular contact and thereby assembled into fibrils (Figure 4) [110]. TDP-43_307–319_ fibrils were able to seed other TDP-43 peptides, suggesting TDP-43 fragments induced other unstructured peptides into fibrils. In addition, TDP-43 redistribution in cells and neurite fragmentation were observed after TDP-43_307–319_ peptide treatment, indicative of the neurotoxicity of the LCD fragments (Figure 4) [110]. Another shorter fragment, TDP-43_311–320_, recognized by Saini et al., presented amyloid properties in vitro, and also had great impact on the aggregation nucleation of the LCD [111].

Apart from the D1 segment, the glutamine (Q)/asparagine (N)-rich segment in TDP-43 LCD is also disordered (Figure 1). Budini et al. demonstrated TDP-43 bound to polyglutamine aggregates via its Q/N-rich segment (TDP-43_342–366_), suggesting Q/N-rich segment in TDP-43 contained amyloid properties [112]. He et al. also identified TDP-43_331–360_ fragment of 40% Q/N content, assembled into amyloid fibrils after incubation (Figure 4) [113]. More importantly, the resulting fibrils induced the ectopically expressed full-length TDP-43 protein aggregation in the cultured cells upon microinjection. As reported by Mompeán et al., another fragment rich in Q/N residues, TDP-43_341–357_, manifested a transition from random coil to β-hairpin, packed into β-sheet-rich amyloid fibrils (Figure 4) [114]. At the same time, they indicated the TDP-43_341–357_ fragment was A11-positive (an antibody that recognizes amyloid oligomer), which revealed the presence of amyloid oligomers from LCD fragments [114]. Interestingly, Jiang et al. reported that the hydrophobic patch in TDP-43 LCD (TDP-43_318–343_), aside from promoting TDP-43 dimerization and mediating the LLPS, also formed amyloid fibrils and seeded TDP-43 proteins into inclusions (Figure 4) [115]. On the contrary, deletion or mutations in this region (TDP-43_318–343_) reduced the aggregation propensity of the LCD, evincing the essential role of this hydrophobic patch in the formation of inclusion bodies [115]

Though it is currently in agreement that the fragments out of the LCD form amyloid fibrils, whether the entire LCD may also assemble into amyloid fibrils remained elusive until recently. In 2021, Fonda et al. evidenced that the LCD formed amyloid fibrils with a β-strand signature and indicated TDP-43_365–400_ was involved in formation of both liquid droplets and amyloid fibrils (Figure 4) [37]. Shenoy et al. and Chang et al. also reported the structure of the LCD fibrils determined by NMR [116,117], and pointed out that the TDP-43_368–414_ fragment was crucial for the formation of the LCD fibrils. (Figure 4) Furthermore, the atomic structures of the fragments and the entire length of the LCD was solved by cryo-EM recently, which provided structural insights for further pathological investigations (Figure 2) [34,105,118]. In 2021, Li et al. demonstrated the entire domain of the LCD assembled into fibrils at pH 4, a mildly acidic condition that allows more dispersed distribution for a better analysis under cryo-EM (Figure 4) [34]. From the reconstructed structure, the twisted left-handed helix structure of fibrils was composed of a tightly packed layer and planar core. This core was made up of 14 β-strands, and the hydrophobic interactions of the side chain between different layers helped stabilize the twisted fibrillar structure. It is worth mentioning that the detailed cryo-EM structures of the entire LCD were different from those of shorter LCD segments (TDP-43_271–313_ and TDP-43_314–353_) reported by Cao et al., though they both formed left-hand helix fibrils (Figure 4) [105]. Li et al. surmised the aforementioned phenomena resulted from the difference in the intramolecular interactions between the entire LCD and its segments. Nevertheless, one needs to take note of the effect that varied pH values may cause in these studies.

Although either fragments or the entire domain of TDP-43 has been confirmed to adopt a β-sheet structure and consequently form amyloid fibrils, the mechanistic studies on the amyloid fibrils’ formation process are less clear thus far. Babinchak et al. showed that TDP-43 LCD could form fibrils in either pH 4 or pH 6 environments with different lag phases. Moreover, they demonstrated the decrease of net charge and the increase of salt concentration enabled faster amyloidogenesis from liquid droplets [71] Until now, growing attention has been drawn to delineate the detailed transition process from liquid droplets to amyloid fibrils. Within these cases, it has been shown that TDP-43 LCD could assemble into amyloid fibrils from the liquid droplets by applying atomic force microscopy [71] These liquid droplets promoted their fibrillization with a shorter lag phase irrespective of electrostatic repulsion. Fonda et al. also showed the three β-strands segments in the TDP-43_365–400_ region stabilized the aged liquid droplets of TDP-43 LCD [37]. Since the formation of TDP-43 LCD fibrils mainly relied on the packing of β-sheets, the tight alignment of the β-strand in TDP-43_365–400_ might serve as a hydrophobic patch to trigger the formation of fibrils. However, the detailed correlation between secondary structural transformation, protein–protein interactions, and environmental factors remain poorly understood.

Another hypothesis proposed by Guenther et al. helps explain how genetic mutations affect the structural transformation from the membraneless organelles to amyloid aggregates [118]. Through the prediction by ZipperDB (ranked by the predicted Rosetta energy) [119], they identified six “steric zippers” that formed irreversible pathogenic amyloids fibrils and four “low-complexity aromatic-rich kinked segments” (LARKs) that formed reversible aggregates [118]. The pathologically phosphorylated mutant segment TDP-43_312–317_ (A315pT) showed higher stability than the wild-type segment, demonstrating the possible pathogenic transformation from reversible LARKs to irreversible fibrils. Furthermore, the protein–protein interactions might also be involved in mediating the phase transition, as proposed by Bhopatkar et al [75,120]. The cleavage products of progranulin, an ALS-associated protein, could interact with TDP-43 LCD and thereby trigger the morphological change toward liquid droplets or amyloid fibrils [75]. One of the cleavage products, granulins-5, promoted the liquid droplet formation of TDP-43 LCD along with RNA; while another cleavage product, granulins-3, mediated the insoluble inclusion formation [75]. Interestingly, the droplets exhibited enhanced staining of Thioflavin S, an amyloid-binding dye, suggesting the internal architecture of liquid droplets may cause the rotational immobilization of dyes [75]. This observation is in line with the work from Fonda et al. that aged liquid droplets were built by stacks of β-strands, hinting at the structural similarity between the liquid droplets and amyloid fibrils. Altogether, these studies have suggested the close correlation between TDP-43 LCD droplets and the pathogenic amyloid fibrils.

### 3.3. The Possible Toxicity of TDP-43 LCD

In 2006, Neumann et al. identified truncated TDP-43 in the inclusions of ALS and FTLD patients [3]. The truncated forms of TDP-43 contained the whole LCD and were insoluble, ubiquitinated, and hyperphosphorylated [3]. Later, the ectopic expression of truncated TDP-43 (either 25 kD or 35 kD variants) readily induced TDP-43 aggregations, which recapitulated the cytotoxicity and disease phenotype in ALS [121,122,123,124,125,126,127]. Currently, some hypotheses have been proposed for the cytotoxicity induced by the LCD. In some cases, the toxicity of the LCD resulted from the impact of membrane disruption [109], endoplasmic reticulum (ER) stress [128], or the sequestration of mRNA-binding proteins [129]. In other cases, it was shown that either the LCD or its fragment could seed the full-length or truncated TDP-43 into aggregates, and induced TDP-43 redistribution from nucleus to cytoplasm [109,110,113,130,131]. These prion-like behaviors of the LCD are toxic in both in vivo and in vitro models. Recently, intercellular (cell-to-cell) propagation of the LCD or its fragment have also been considered as a driver for disease progression [110,129,132]. Until now, the reported pathways to disseminate TDP-43 include: (1) the secretion through exosomes from donor cells [133,134], (2) the propagation by tunneling nanotubes-like structure [134], and (3) the direct transmission across axon terminals [132]. Finally, the full-length TDP-43 formed toxic oligomers both in the cellular models and FTLD patients [132,135,136]. Though the role of TDP-43 oligomers in TDP-43 proteinopathy has been suggested, whether TDP-43 LCD also participates through a similar scenario remains undetermined.

## 4. Concluding Remark

While the LCD is essential for TDP-43 in RNA processing and stress granule assembly, it is also blamed for irreversible pathological aggregation. Currently, a growing body of the recently introduced “liquid-like organelles” opens up a new avenue to the interchangeability among the three faces of the LCD, the soluble form, the liquid droplets, and the insoluble aggregate. Though the LCD is generally considered as unstructured, the unveiling of the helical structure in it and the induced helical–helical contact between the LCD highlight the close correlation between the structure transformation and LLPS. In addition to the LCD, recent studies also suggested the roles of NTD and RRM in TDP43-containing granules. Depending on the binding partners and the subcellular localizations, the LCD of TDP-43 is enrolled in either the stress granules or paraspeckles to accommodate RNA or RNA-binding proteins (e.g., hnRNP, FUS, and G3BP1). In the case of stress granules, since the homotypic and heterotypic interactions between the LCD and its clients are relatively weak, the membraneless organelles rapidly disappear upon the removal of stresses [7]. On the other hand, during the liquid-droplet maturation, the thermodynamically favored beta-strand-rich structure gradually dominates in the process, which resembles the transformation from stress granules to amyloid-like inclusions upon prolonged stress [37]. Nevertheless, due to the intrinsic nature of TDP-43 LCD, either the soluble TDP-43 redistribution or the possible LCD fragmentation may also lead to the formation of amyloid fibrils and inclusion bodies. As the TDP-43 inclusions form under the stress, the propagation of these pathological inclusions may worsen the situation and finally lead to disease. These different scenarios accommodate the roles of TDP-43 LCD in both its functional and pathological states, which is subject to genetic mutation and/or environmental stimuli.

Though accumulating studies have evinced that TDP-43 LCD could self-assemble into liquid droplets or amyloid fibrils at different in vitro conditions, the underlying molecular mechanism remains elusive, and comprehensive studies to delineate the LCD structural transformation from liquid droplets to amyloid fibrils are imperative. Another missing puzzle in this scenario is the unidentified roles of TDP-43 LCD oligomers. Thus, elucidating the correlations between TDP-43 oligomers, liquid droplets, and amyloid fibrils might significantly advance the understanding of TDP-43 proteinopathy. Furthermore, since TDP-43 has also been implicated in other neurodegenerative diseases (e.g., Parkinson’s disease and Alzheimer’s disease) [137], whether TDP-43 LCD also participates in their pathologies requires further investigation. With more and more studies having revealed the TDP-43 LCD morphological transitions, physiological functions, and proteinopathies, we expect these collective results may eventually shed light on the TDP-43 pathogenesis mechanism and benefit the therapeutic development of TDP-43-related diseases.

## Data Availability

The study did not report any data.
